# ﻿First amphibious Crinocheta (Isopoda, Oniscidea) from the Neotropics with a troglobitic status: a relictual distribution

**DOI:** 10.3897/zookeys.1192.114230

**Published:** 2024-02-19

**Authors:** Carlos Mario López-Orozco, Ivanklin Soares Campos-Filho, Livia Medeiros Cordeiro, Jonas Eduardo Gallão, Yesenia M. Carpio-Díaz, Ricardo Borja-Arrieta, Maria Elina Bichuette

**Affiliations:** 1 Laboratório de Estudos Subterrâneos, Universidade Federal de São Carlos, São Carlos, São Paulo, Brazil Universidade Federal de São Carlos São Carlos Brazil; 2 Department of Biological Sciences, University of Cyprus, Lefkosia (Nicosia), Cyprus University of Cyprus Nicosia Cyprus; 3 Instituto Brasileiro de Estudos Subterrâneos, São Paulo, Brazil Instituto Brasileiro de Estudos Subterrâneos São Paulo Brazil; 4 Grupo de Espeleologia Serra da Bodoquena, São Paulo, Brazil Grupo de Espeleologia Serra da Bodoquena São Paulo Brazil; 5 Grupo de Investigación en Biología Descriptiva y Aplicada, Universidad de Cartagena, Programa de Biología, Campus San Pablo, Cartagena de Indias, Colombia Universidad de Cartagena Cartagena de Indias Colombia

**Keywords:** Cave fauna, new genus, new species, Serra da Bodoquena, southwestern Brazil, тerrestrial isopods, troglobitic species

## Abstract

The first freshwater amphibian representative of Crinocheta (Oniscidea) from the Neotropics is described from caves within the Brazilian Cerrado biome, state of Mato Grosso do Sul. *Kadiweuoniscusrebellis***gen. et sp. nov.** is placed in the family Philosciidae. The present work represents a significant contribution to future studies seeking to understand the ecological and evolutionary processes of Crinocheta within the Neotropical region. Moreover, it highlights the importance of biodiversity surveys in subterranean environments toward effective conservation efforts of these unique habitats and their surroundings.

## ﻿Introduction

Terrestrial isopods (Oniscidea) are considered the most diverse group of isopods, with more than 4000 species in more than 500 genera in 38 or 39 families (Schmalfuss 2003; Taiti 2004; Javidkar et al. 2015; Sfenthourakis and Taiti 2015; Dimitriou et al. 2019; Campos-Filho and Taiti 2021). These organisms are distributed in almost all terrestrial environments on the planet, ranging from the supralittoral zone to high mountains, and from tropical zones to deserts (Schmalfuss 2003; López-Orozco et al. 2022). Additionally, a considerable number of species inhabit subterranean environments (Vandel 1973; Schmalfuss 2003; Taiti and Gruber 2008; Bedek et al. 2011; Taiti and Xue 2012; Tabacaru and Giurginca 2013; Reboleira et al. 2015).

Regarding the phylogeny of the group, morphological studies show Oniscidea as monophyletic, including the Sections Ligiidae, Tylidae, Mesoniscidae, Synocheta, and Crinocheta (Schmalfuss 1989; Wägele 1989; Erhard 1998; Schmidt 2008). All these authors recognized Synocheta as the sister group of Crinocheta. The last section is the most diverse, representing more than 80% of the entire suborder inhabiting various types of habitats (Schmidt 2002, 2003, 2008; Schmalfuss 2003). Recent molecular evidence has revealed that the genus *Ligia* Fabricius, 1798, is closer to marine groups of isopods, which raises doubts about the monophyly of Oniscidea (Lins et al. 2017; Dimitriou et al. 2019). However, future studies with integrative approaches will be necessary to clarify the phylogenetic relationships of the group.

In the last two decades, studies of terrestrial isopods have increased globally (Vittori and Dominko 2022). To date, Brazil holds the highest diversity of species in the Neotropical region, comprising more than 250 species (see Campos-Filho et al. 2018, 2019, 2020; Cardoso et al. 2020a, 2020b, 2021). Among them, more than 40 are considered obligatory cave-dwellers (troglobites), grouped in the families Armadillidae, Philosciidae, Pudeoniscidae, Scleropactidae and Styloniscidae (Campos-Filho et al. 2018, 2019, 2020, 2022a, 2022b, 2022c, 2023; Cardoso et al. 2020a, 2020b, 2021; Cardoso and Ferreira 2023; López-Orozco et al. in press). Moreover, Styloniscidae comprise the highest number of troglobitic species, some of which have amphibious habits (e.g., *Xangoniscus* spp. and *Spelunconiscus* spp.) (Campos-Filho et al. 2014, 2022a; Bastos-Pereira et al. 2017, 2022; Cardoso et al. 2020a).

Most of the amphibian species of Oniscidea found in caves belong to the Section Synocheta, primarily from the families Styloniscidae and Trichoniscidae (Vandel 1973; Taiti and Gruber 2008; Bedek et al. 2011; Taiti and Xue 2012; Tabacaru and Giurginca 2013; Campos-Filho et al. 2014, 2019, 2022a, 2022b, 2022c; Souza et al. 2015; Cardoso et al. 2020a, 2021; Bastos-Pereira et al. 2022; Reboleira et al. 2015). In Crinocheta, this type of habit has been described in the family Olibrinidae, which includes species found in caves and marine littoral environments in the genera *Castellanethes* Brian, 1952, *Olibrinus* Budde-Lund, 1912 and *Paradoniscus* Taiti & Ferrara, 2004 (Taiti and Ferrara 2004; Taiti and Gardini 2022; Moutaouakil et al. 2023). Regarding the American continent, there is only the record of *Olibrinusantennatus* (Budde-Lund, 1902) in the marine coast of the state of Rio Grande do Norte, Brazil, inhabiting mangrove swamps and under coral rock in the coastal environment (Araujo and Taiti 2007; Campos-Filho et al. 2018). In the family Philosciidae, this habit is present in some representatives of *Haloniscus* Chilton, 1920 from Australia (Taiti and Humphreys 2001; Guzik et al. 2019; Stringer et al. 2019), and epigean species *Androdelosciatarumae* (Lemos de Castro, 1984) in the Central Amazon (Warburg et al. 1997). This amphibious habit has been considered as a secondary condition that appeared several times within Oniscidea (Schmidt 2008; Taiti and Xue 2012; Taiti et al. 2018; Sfenthourakis et al. 2020).

In the present study, a freshwater amphibian representative of Crinocheta (Philosciidae) with troglobitic status is described for the first time in the Neotropical region. *Kadiweuoniscusrebellis* gen. et sp. nov. is described from caves in the Brazilian Cerrado biome, state of Mato Grosso do Sul, Serra da Bodoquena karst area.

## ﻿Materials and methods

### ﻿Study area

**Figure 1.**
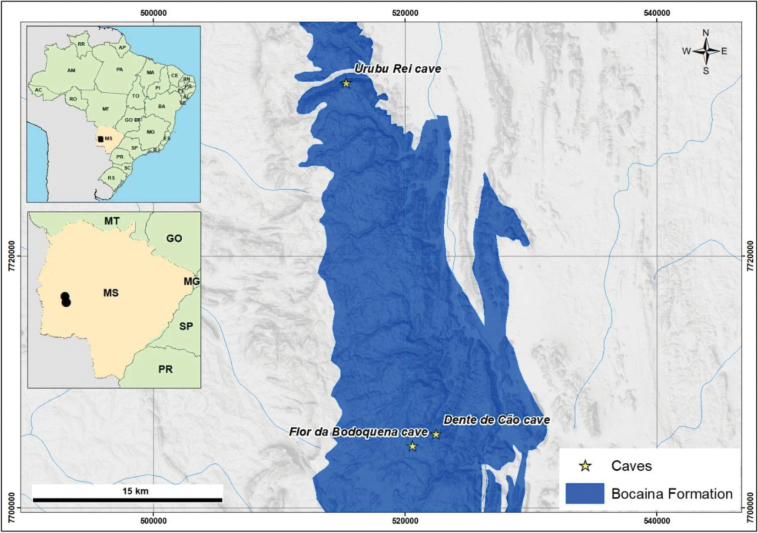
Map of the study area and distribution of *Kadiweuoniscusrebellis* López-Orozco, Campos-Filho & Bichuette gen. et sp. nov. in the Serra da Bodoquena, Mato Grosso do Sul.

**Figure 2.**
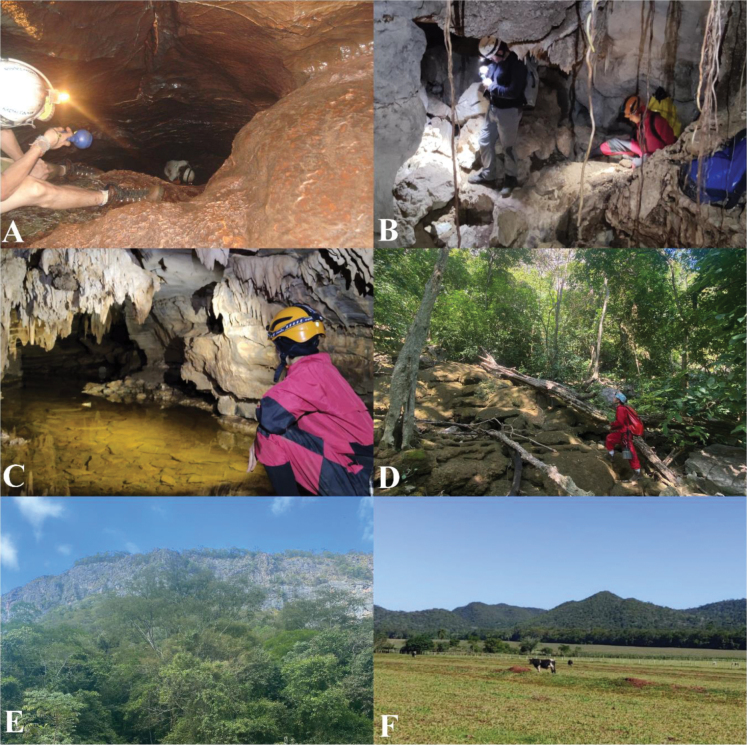
Study area: **A** Flor da Bodoquena Cave **B** Urubu Rei Cave **C** Dente de Cão Cave **D** epigean environment in Flor da Bodoquena Cave **E** limestones in the Serra da Bodoquena karst area **F** livestock and agriculture in the Serra da Bodoquena.

The material was collected from three limestone caves of Serra da Bodoquena karst area, located in the Bodoquena municipality, state of Mato Grosso do Sul, southwestern Brazil (Figs 1, 2). The caves occupy an area about 220 km N to S and may reach 40 km E to W, encompassing many flooded caves beside a few sparse limestone hills, located about 100 km N to W along the Paraguay Belt. The area belongs to the Corumbá (geomorphological) Group, Bocaina Formation, and it is classified as having an Aw (tropical) climate, characterized by a dry winter and humid summer (Bedek et al. 2018, 2020). It is known for its high diversity of troglobites (Camargo and Lourenção 2007; Cordeiro et al. 2014), surpassing 34 species, many of them aquatic (Trajano et al. 2016). The native vegetation in the area consists of savanna in contact with semi-deciduous seasonal forest, within the Cerrado Biome (Galati et al. 2003; Boggiani et al. 2011). Two of the caves are located within the Serra da Bodoquena National Park (PNSB, Parque Nacional Serra da Bodoquena), a National Conservation Unit created in 2000 that covers an area of 76,481 hectares and contains numerous caves (Camargo and Lourenção 2007; Lobo 2007; Cordeiro et al. 2014). Currently, livestock is the main economic activity in the region, followed by tourism, including speleotourism. The latter has grown in economic importance for the municipalities of Bonito, Bodoquena and Jardim (Lobo 2007; Cordeiro et al. 2014).

### ﻿Collections and taxonomy

Specimens were collected by active search with the aid of tweezers and brushes, and stored in 75% ethanol; microhabitat data was also recorded. The identifications were based on morphological characters with the use of micropreparations in Hoyer’s medium (Anderson 1954). The illustrations were made with the aid of a camera lucida mounted on a Zeiss Stemi SV6 stereomicroscope and Leica DMLS microscope. The final illustrations were prepared using the software GIMP v.2.8 with the method proposed by Montesanto (2015, 2016). For scanning electron microscopy (SEM), two individuals were used, one male and one female, without performing dissections. The specimens were dried using Critical Point Drying and mounted on a plastic sheet. Uncoated SEM preparations were examined using an FEI Quanta 250 (at the UFSCar). The figures were edited using GIMP v.2.8.

The material examined is deposited in the zoological collection of the Labo­ratório de Estudos Subterrâneos (LES), Universidade Federal de São Carlos, São Carlos, Brazil (curator: Maria E. Bichuette).

## ﻿Systematic account

### ﻿Suborder Oniscidea Latreille, 1802

**Family Philosciidae Kinahan, 1857**

#### ﻿Genus Kadiweuoniscus

Taxon classificationAnimaliaIsopodaPhilosciidae

López-Orozco, Campos-Filho & Bichuette gen. nov.

9E8A6E5F-9376-5C38-8877-30072BEB1B03

https://zoobank.org/C1386E76-7F74-45AA-9097-C7A757FD39C3

##### Type species.

*Kadiweuoniscusrebellis* López-Orozco, Campos-Filho & Bichuette sp. nov., by present designation and monotypy.

##### Diagnosis.

Troglobitic species with amphibious habit; animals about 5 mm long; dorsal surface weakly granulated; *noduli laterales* absent; cephalon with lateral lobes weakly developed, frontal and suprantennal lines absent; pleonites 3–5 with epimera elongated, forming acute tips; telson triangular; antennula of three articles, distal article separated from medial article by fine suture; antenna with flagellum of three articles, apical organ long; molar penicil of mandibles dichotomized; maxillule outer branch with eight teeth; maxilla bilobate; maxilliped endite without penicil; male pereopods 1–7 gradually elongated; dactylar seta short and simple; uropod endopod inserted slightly proximally; pleopods 3–5 exopods with fringe of thin setae on all margins.

##### Etymology.

The new genus is named after the Kadiwéu indigenous people. The Kadiwéu are known as “Indian riders”, due to their horse-riding prowess, keeping in their mythology, art and rituals the way of being of a hierarchical society between masters and captives.

##### Remarks.

The family Philosciidae comprises more than 600 species in 113 genera widely distributed in Australia, southern Asia, Africa, Europe and the Americas (Leistikow 2001; Schmalfuss 2003; Boyko et al. 2023). To date, the family is considered paraphyletic due to characteristics shared with the Halophilosciidae and Scleropactidae (Leistikow 2001; Schmidt 2003, 2008).

The family has great morphological plasticity and the representatives are mainly recognized by the ‘runner-type’ habitus (sensu Schmalfuss 1984), body with a dorsal surface smooth or slightly tuberculated, pereonites 1–7 with one or two lines of *noduli laterales* per side (sometimes present on cephalon and pleonites), antennula and antennal flagellum of three articles, mandibles with molar penicil simple or dichotomized, maxillula outer endite with outer set of teeth simple or cleft or pectinated, maxilla bilobated, maxilliped endite bearing ventral penicil or triangular seta (sometimes absent), uropod branches unequal or similar in length and inserted at same or on distinct levels, and pleopod exopods with out respiratory areas or with covered monospiracular lungs (Taiti and Ferrara 1980, 1982; Ferrara et al. 1994; Araujo and Leistikow 1999; Leistikow and Araujo 2001; Leistikow 2001).

*Kadiweuoniscus* gen. nov. is included in Philosciidae by having most of these mentioned characters. The new genus is easily distinguishable from the other genera of Philosciidae due to its amphibian habit, and the pleonites 3–5 epimera elongated. As mentioned, the amphibious behavior is also present in species of *Haloniscus*; however, the new genus differs in the cephalon lacking frontal and suprantennal lines (vs. present in *Haloniscus*, except *H.anophthalmus* Taiti, Ferrara & Iliffe, 1995), pleonites 3–5 epimera elongated (vs. pleonites 3–5 epimera reduced in *Haloniscus*), antennula distal and medial articles separated by fine suture (vs. antennula with three distinct articles in *Haloniscus*), antennal flagellum with long apical organ (vs. short in *Haloniscus*), maxillula outer branch with 4 + 4 teeth, long and curved (vs. maxilla with 4 or 5 + 6 in *Haloniscus*), and maxilliped endite without penicil (vs. present in *Haloniscus*).

#### ﻿ Kadiweuoniscus rebellis

Taxon classificationAnimaliaIsopodaPhilosciidae

López-Orozco, Campos-Filho & Bichuette sp. nov.

22A2978B-19C5-5179-8C21-3D214D34847F

https://zoobank.org/F5A2614F-20F7-44CC-865F-C2A16F2329D1

**Figure 3.**
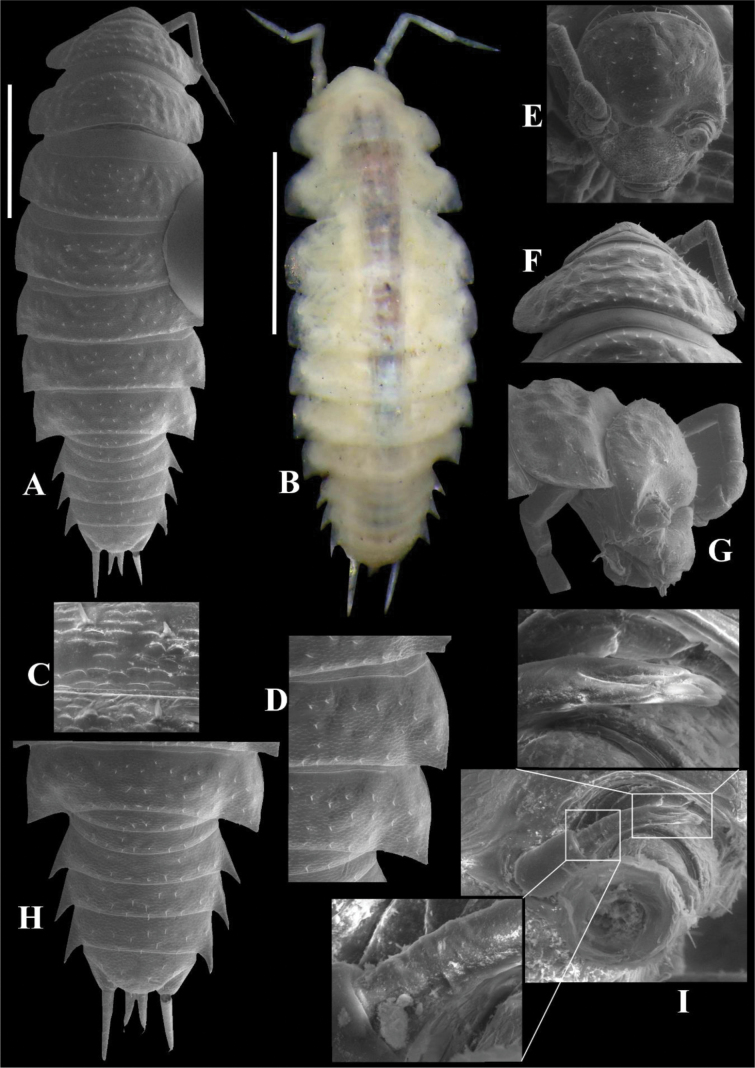
*Kadiweuoniscusrebellis* López-Orozco, Campos-Filho & Bichuette gen. et sp. nov. (♀ paratype, LES 0029050) **A, B** habitus, dorsal view **C** dorsal scale-seta **D** epimera 6–7 **E** cephalon, frontal view **F** cephalon and pereonite 1, posterior view **G** cephalon and pereonite 1, lateral view **H** pleon, telson and uropods **I** antennula. Scale bars: 1 mm.

**Figure 4.**
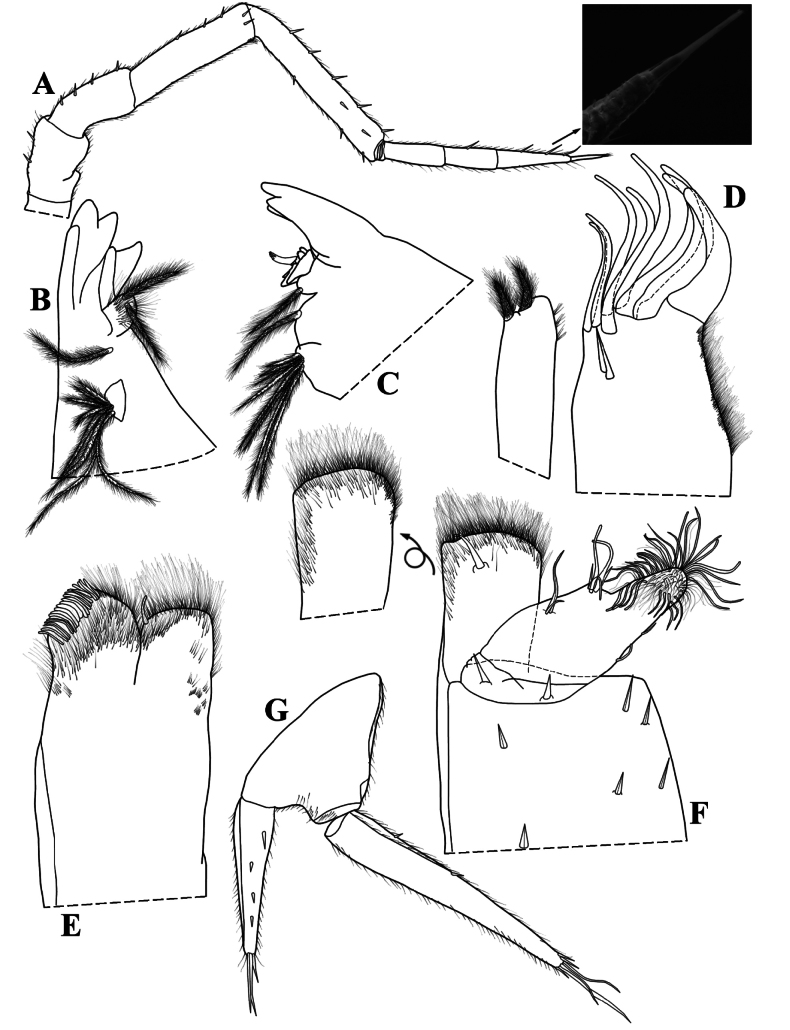
*Kadiweuoniscusrebellis* López-Orozco, Campos-Filho & Bichuette gen. et sp. nov. (♀ paratype, LES 0029049) **A** antenna, with flagellum detail **B** left mandible **C** right mandible **D** maxillula **E** maxilla **F** maxilliped, arrow illustrating the endite in caudal view **G** uropod.

**Figure 5.**
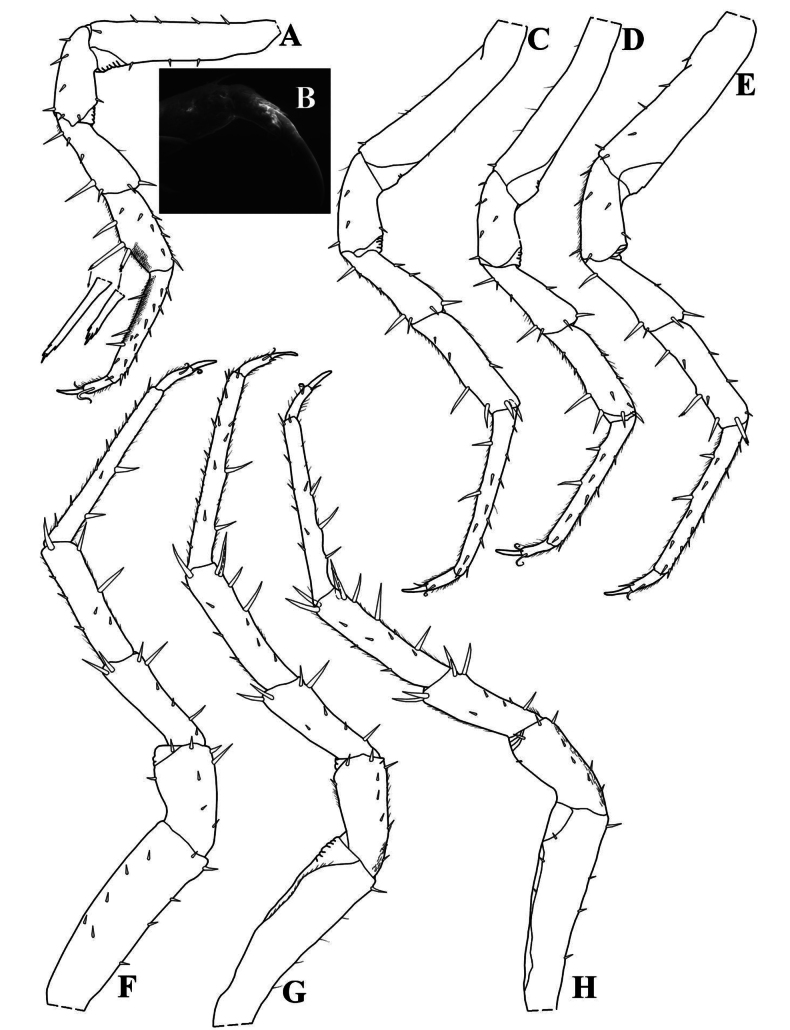
*Kadiweuoniscusrebellis* López-Orozco, Campos-Filho & Bichuette gen. et sp. nov. (♂ holotype, LES 0029048) **A** pereopod 1 **B** dactylus in rostral view; (♂ paratype, LES 0029049) **C** pereopod 2 **D** pereopod 3 **E** pereopod 4 **F** pereopod 5 **G** pereopod 6 **H** pereopod 7.

**Figure 6.**
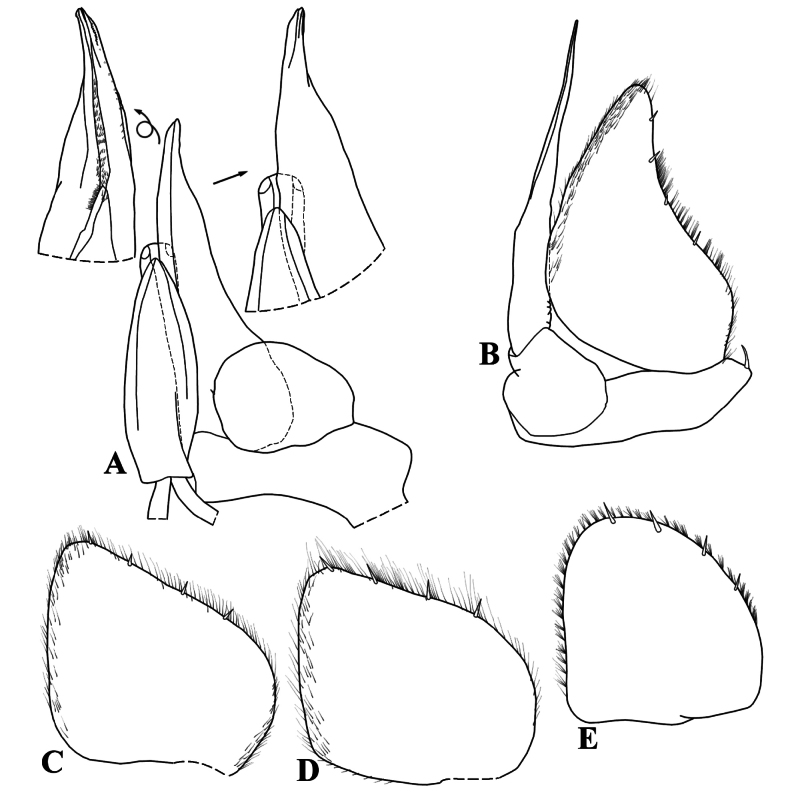
*Kadiweuoniscusrebellis* López-Orozco, Campos-Filho & Bichuette gen. et sp. nov. (♂ paratype, LES 0029049) **A** genital papilla and pleopod 1 **B** pleopod 2 **C** pleopod 3 exopod **D** pleopod 4 exopod **E** pleopod 5 exopod.

**Figure 7.**
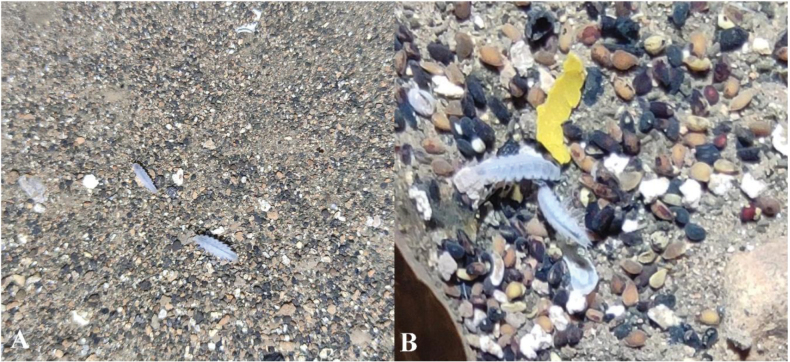
**A** and **B***Kadiweuoniscusrebellis* López-Orozco, Campos-Filho & Bichuette gen. et sp. nov. submerged on rocky substrate with silt and pebbles.

Figs 1 , 2 , 3 , 4 , 5 , 6 , 7 , Suppl. material 1

##### Type material.

Brazil ● 1♂, ***holotype***, Flor da Bodoquena Cave, Bodoquena, state of Mato Grosso do Sul, 20°45'19"S, 56°48'8"W, 14.VIII.2011, leg. LM Cordeiro, LES 0029048 ● 1♂, 1♀ (part in micropreparations), ***paratypes***, Dente de Cão Cave, 20°44'48"S, 56°47'4.2"W, 13.VI.2022, leg. LM Cordeiro, A Chagas-Jr, ME Bichuette, LES 0029049 ● 2♀♀, ***paratypes***, same data as previous, LES 0029050 ● 1♀, ***paratypes***, same data as holotype, LES 0029051 ● 1♀, ***paratypes***, Urubu Rei Cave, 20°29'40"S, 56°51'11"W, 16.VI.2022, leg. LM Cordeiro, A Chagas-Jr, ME Bichuette, LES 0029052 ● 1♀, ***paratypes***, same data as previous, LES 0029053 ● 1♂, same data as previous, LES 0029054.

##### Description.

Maximum body length: male 4.5 mm, female 5 mm. Body outline as in Fig. 3A, B. Colourless (Fig. 3B). Dorsal surface granulated bearing pointed scale-setae (Fig. 3A, C, D). Cephalon (Fig. 3E–G) with small semicircular antennary lobes; eyes absent. Pereonites 1–2 with epimera semicircular, 3–7 with posterior corners gradually more acute (Fig. 3A, B, D, F, G). Pleon (Fig. 3H) narrower than pereon, pleonite 3–5 epimera elongated and acute. Telson (Fig. 3H) broader than long, lateral sides almost straight, rounded apex. Antennula (Fig. 3I) distal article longest with four apical aesthetascs. Antenna (Fig. 4A) long, not surpassing pereonite 3 when extended backwards; flagellum of articles subequal in length; apical organ shorter than basal article of flagellum, bearing small free sensilla. Mandibles with molar penicil of six to seven branches; right mandible (Fig. 4B) with 1+1 free penicils; left mandible (Fig. 4C) with 2+1 free penicils. Maxillula (Fig. 4D) inner endite bearing two setose penicils, distal margin rounded; outer endite with 4 + 4 teeth simple, elongated and curved. Maxilla (Fig. 4E) with setose lobes; outer lobe slightly smaller than inner lobe, quadrangular and covered with thin and long setae; inner lobe rounded and covered with thin and thick setae. Maxilliped (Fig. 4F) basis rectangular; first article of palp bearing two setae; endite rectangular, medial seta overpassing distal margin, ventrally with setose sulcus. Uropod (Fig. 4G) protopod and exopod grooved on outer margin. Pereopods 1–7 bearing sparse setae on sternal margin. Pereopod 1 (Fig. 5A) carpus with antennal grooming brush reduced, composed by short scale-setae; dactylus with ungual and dactylar setae simple (Fig. 5B). Pleopod exopods without respiratory areas.

**Male.** Pereopod 1–7 (Fig. 5A–H) gradually more elongated, without particular modifications. Genital papilla (Fig. 6A) with lanceolate ventral shield; papilla longer than ventral shield bearing two subapical orifices. Pleopod 1 (Fig. 6A) exopod ovoidal, inner margin with one small seta; endopod stout, three times longer than exopod, slightly bent outwards, apex bearing setae on inner margin. Pleopod 2 (Fig. 6E) exopod triangular, outer margin concave bearing four setae; endopod flagelliform, slightly longer than exopod. Pleopod 3 and 4 (Fig. 6C, D) exopods rhomboid, outer margin with four setae, inner margin slightly convex. Pleopod 5 (Fig. 6E) exopod rhomboid, longer than wide, distal and outer margins rounded bearing four small setae.

##### Etymology.

The new species name alludes to the resistance group from the Star Wars fiction series, the Rebel Alliance, that fights against the Empire. The Kadiwéu indigenous people were known as warriors, and they fought for Brazil in the Paraguayan War to reclaim and secure their lands in the Serra da Bodoquena region. Today, they are confined to the outskirts of the Bodoquena plateau and the Pantanal plain. The designation ‘rebellis’ is used as an adjective for the genus name.

##### Ecological remarks.

The physicochemical data of microhabitats of *Kadiweuoniscusrebellis* gen. et sp. nov. are: pH = 7.5, high conductivity (c. 0.450 µS.cm^-1^), moderate temperature (22 °C) and moderate dissolved oxygen (ca. 6.0 mg.l^-1^). pH values (neutral to basic) are typical of karst waters. The abundance is particularly low in each cave, and they have a preference for rocky substrates with a silty and pebble bottom (Fig. 7A, B). Amphibious habit (see Suppl. material 1).

## ﻿Discussion

The Serra da Bodoquena karst area exhibits taxonomic singularities among invertebrates, housing some remarkable phylogenetic relicts among peracarid crustaceans, such as *Potiicoarabrasiliensis* Pires, 1987 (Spelaeogriphacea) and *Megagidiellaazul* Koenemann & Holsinger, 1999 (Amphipoda) (Pires 1987; Koenemann and Holsinger 1999). To date, no records of such singularities within Isopoda, particularly Oniscidea, have been documented. Cordeiro et al. (2014) reported a low diversity of terrestrial isopods in this region. At present, the records include *Circoniscusintermedius* Souza & Lemos de Castro, 1991 and *Diploexochuscarrapicho* Campos-Filho, López-Orozco & Taiti (Campos-Filho et al. 2014, 2023). The discovery of a new freshwater amphibian genus highlights the taxonomic significance to both Serra da Bodoquena karst areas and the Neotropical region. Moreover, *K.rebellis* gen. et sp. nov. represents a significant addition to our ecological knowledge of Neotropical Philosciidae, considering its amphibious habit. It is worth noting that the majority of species within this family commonly inhabit the edaphofauna in the epigeal environment (Leistikow 2001).

*Kadiweuoniscusrebellis* gen. et sp. nov. exhibits several distinctive features, including troglomorphisms, such as the absence of eyes and body pigments, as well as the elongation of the pereiopods. These characteristics are typical traits found in amphibious troglobitic Oniscidea (Campos-Filho et al. 2014, 2019, 2022a, 2022b; Souza et al. 2015; Cardoso et al. 2020a, 2020b, 2021; Bastos-Pereira et al. 2022). One key trait common among representatives with this type of habit is the absence of *noduli laterales*, which represents an important character in the classification of some groups of terrestrial isopods (Schmidt 2008). This characteristic is observed in several genera with epigean species within Philosciidae (e.g., *Oxalaniscus* Leistikow, 2000, *Ischioscia* Verhoeff, 1928, *Parischioscia* Lemos de Castro, 1967, *Mirtana* Leistikow, 1997, *Pentoniscus* Richardson, 1913, *Yaerikima* Leistikow, 2001, *Formicascia* Leistikow, 2001, *Roraimoscia* Leistikow, 2001, *Oreades* Vandel, 1968 and *Ecuadoroniscus* Vandel, 1968 (Leistikow 2001). However, the significance of this character remains unknown in many fields of biology, and further research is needed to elucidate its usefulness (Leistikow 2001; Schmidt 2008).

Based on the phylogeny of South American Philosciidae, the genera *Quintanoscia* Leistikow, 2000 and *Oxalaniscus* were recovered as basal groups (Leistikow 2001). These two genera show typical characters of the groundpattern Oniscidea, such as a subrectangular maxilla, the maxilliped palp with prominent setal tufts and the endite with a prominent penicil (Leistikow 2000, 2001). *Kadiweuoniscus* gen. et sp. nov. shares the first two characteristics, however, it lacks the penicil in the endite of the maxilliped, which may suggest it is basal within Philosciidae. Further investigations including molecular data are necessary to clarify the position of the new genus within the family. Our discovery marks the initial documentation of the troglobitic amphibian Crinocheta in the Neotropics, serving as a pivotal link for comprehending aspects related to the biology, ecology, morphology, distribution and evolution of Philosciidae.

The Flor da Bodoquena Cave is a vertical fracture (Fig. 2A), with the water reaching 20 m deep and extending only 150 meters. Specimens of *K.rebellis* gen. et sp. nov. were found in the walls of the cave. The fracture ends at the level of the sheet, where the stygobitic catfish *Trichomycterusdali* Rizzato, Costa, Trajano & Bichuette, 2011 is also recorded, possibly a predator of the isopods. During the rainy season, the current increases and the water level rises, oscillating between 10 and 12 m. Dispersion of the species possibly follows this oscillation. This cave is at a higher altitude compared to the other two where the species occurs (Urubu Rei and Dente de Cão, Fig. 2B–D), and may represent the source population. Urubu Rei and Dente de Cão caves have conduits with lentic waters and bottoms formed by silt, pebbles and rocky substrate (Fig. 2B, Urubu Rei Cave). In the case of Urubu Rei, the water rises drastically in the rainy season, becoming extremely lotic, which also could disperse/spread the isopods; the same is not observed for Dente Cão Cave.

Considering the species distribution, although the limestones are continuous at the surface (Fig. 2E), hydrological barriers have been formed due to fluvial incisions that, in the north plateau, have cut down to non-carbonate rocks, forming compartments corresponding to micro-basins (Cordeiro et al. 2014). The species occurs in the upper part of these compartments, and its distribution in the three caves could be explained by one colonization event and, after the regional uplifting prior to the Pliocene/Pleistocene transition, raising the area to altitudes of 1000 m, followed by the current subsidence responsible for the formation of the adjacent Pantanal Basin. The three populations may be isolated by geological processes, as suggested by Moracchioli and Trajano (2002) for *P.brasiliensis*. These facts, allied with the marked troglomorphisms and unique characteristics, could indicate a relictual occupation of the Serra da Bodoquena subterranean habitats.

### ﻿Conservation remarks

The species occurs in caves within the boundaries of the Serra da Bodoquena National Park, and is, a priori, legally protected. However, the caves are not controlled or even inspected by governmental bodies dealing with tourism and visitors. Furthermore, their surroundings have deforested areas with livestock farming and agriculture activities (Fig. 2F), which drastically reduces the quality of the habitat. These facts make the species vulnerable in relation to its effective protection. Ecological monitoring projects can help in the understanding of the distribution and singularities of the species, which would assist in conservation efforts. The restricted distribution makes the species vulnerable and potentially endangered.

Finally, considering that specimens of the taxon described here have been collected since 2011, it is essential to emphasize the significance of scientific collections in the study of biodiversity. These collections serve as repositories of historical information about natural environments. Therefore, it becomes crucial to review the materials collected over extended periods and stored in such institutions. By doing so, we can enhance our understanding of the vast taxonomic diversity present in our ecosystems. Scientific collections play a crucial role in research, allowing the examination of specimens over time and across different locations, and safeguarding our biodiversity. Without these collections, our knowledge of the natural world and the species it harbors would be severely limited.

## Supplementary Material

XML Treatment for Kadiweuoniscus

XML Treatment for Kadiweuoniscus rebellis

## ﻿Additional information

### Author ORCIDs

Carlos Mario López-Orozco https://orcid.org/0000-0002-3251-7739

Ivanklin Soares Campos-Filho https://orcid.org/0000-0001-6139-8241

Livia Medeiros Cordeiro https://orcid.org/0000-0002-0742-897X

Jonas Eduardo Gallão https://orcid.org/0000-0002-5268-7946

Yesenia M. Carpio-Díaz https://orcid.org/0000-0001-5116-9736

Ricardo Borja-Arrieta https://orcid.org/0000-0002-5064-5080

Maria Elina Bichuette https://orcid.org/0000-0002-9515-4832

### Data availability

All of the data that support the findings of this study are available in the main text or Supplementary Information.

## Supplementary materials

Supplementary material 1 Supplementary video Data type mp4 Explanation note *Kadiweuoniscusrebellis* López-Orozco, Campos-Filho & Bichuette gen. et sp. nov. locomotor behavior in the Flor da Bodoquena Cave, in rocky substrate with silt and pebbles. Video on YouTube: https://youtube.com/shorts/bNqp97PdPcY?feature=share. https://binary.pensoft.net/file/986317This dataset is made available under the Open Database License (http://opendatacommons.org/licenses/odbl/1.0/). The Open Database License (ODbL) is a license agreement intended to allow users to freely share, modify, and use this Dataset while maintaining this same freedom for others, provided that the original source and author(s) are credited.Carlos Mario López-Orozco, Ivanklin Soares Campos-Filho, Livia Medeiros Cordeiro, Jonas Eduardo Gallão, Yesenia M. Carpio-Díaz, Ricardo Borja-Arrieta, Maria Elina Bichuette

## References

[B1] Anderson LE (1954) Hoyer’s solution as a rapid permanent mounting medium for bryophytes. The Bryologist 57(3): e242. 10.1639/0007-2745(1954)57[242:HSAARP]2.0.CO;2

[B2] Araujo PB Leistikow A (1999) Philosciids with pleopodal lungs from Brazil, with description of a new species (Crustacea, Isopoda). Contributions to Zoology (Amsterdam, Netherlands) 68(2): 109–141. 10.1163/18759866-06802004

[B3] Araujo PB Taiti S (2007) Terrestrial isopods (Crustacea, Oniscidea) from Rocas Atoll, northeastern, Brazil. Arquivos do Museu Nacional. Museu Nacional (Brazil) 65: 347–355.

[B4] Bastos-Pereira R Souza LA Ferreira RL (2017) A new amphibious troglobitic styloniscid from Brazil (Isopoda, Oniscidea, Synocheta). Zootaxa 4294(2): 292–300. 10.11646/zootaxa.4294.2.11

[B5] Bastos-Pereira R Souza LA Sandi BDS Ferreira RL (2022) A new species of *Spelunconiscus* (Isopoda: Oniscidea: Styloniscidae) for Brazilian caves: new record for the type species and an emended diagnosis for the genus. Nauplius 30: e2022018. 10.1590/2358-2936e2022018

[B6] Bedek J Taiti S Gottstein S (2011) Catalogue and atlas of cave-dwelling terrestrial isopods (Crustacea: Oniscidea) from Croatia. Natura Croatica 20(2): 237–354. https://hrcak.srce.hr/75047

[B7] Bedek HE Zimmermann NE McVicar TR Vergopolan N Berg A Wood EF (2018) Present and future Köppen-Geiger climate classification maps at 1-km resolution. Scientific Data 5(1): e180214. 10.1038/sdata.2018.214 PMC6207062 30375988

[B8] Bedek HE Zimmermann NE McVicar TR Vergopolan N Berg A Wood EF (2020) Publisher Correction: Present and future Köppen-Geiger climate classification maps at 1-km resolution. Scientific Data 7(1): e274. 10.1038/s41597-020-00616-w PMC7431407 32807783

[B9] Boggiani PC Trevelin AC Sallun-Filho W Oliveira EC Almeida LHS (2011) Turismo e conservação de tufas ativas da Serra da Bodoquena, Mato Grosso do Sul. Pesquisas em Turismo e Paisagens Cársticas 4: 55–63.

[B10] Boyko CB Bruce NL Hadfield KA Merrin KL Ota Y Poore GCB Taiti S (2023) [Onwards]Philosciidae Kinahan, 1857. Accessed by World Marine, Freshwater and Terrestrial Isopod Crustaceans database. https://www.marinespecies.org/aphia.php?p=taxdetails&id=246825 [Accessed on 10^th^ April, 2023]

[B11] Camargo RR Lourenção MLF (2007) Levantamento espeleológico da Serra da Bodoquena. In: Brazilian Society of Speleology (Ed.) Proceedings of the 29th Congresso Brasileiro de Espeleologia, July 2007, Ouro Preto.SEE/SBE, Ouro Preto, 1–7.

[B12] Campos-Filho IS Taiti S (2021) Oniscidea taxonomy: present and future. Abstract book of the 11^th^ International Symposium on Terrestrial Isopod Biology. Spinicornis, Ghent, 9. https://spinicornis.be/istib2021/presentations/

[B13] Campos-Filho IS Araujo PB Bichuette ME Trajano E Taiti S (2014) Terrestrial isopods (Crustacea: Isopoda: Oniscidea) from Brazilian caves. Zoological Journal of the Linnean Society 172(2): 360–425. 10.1111/zoj.12172

[B14] Campos-Filho IS Cardoso GM Aguiar JO (2018) Catalogue of terrestrial isopods (Crustacea, Isopoda, Oniscidea) from Brazil: An update with some considerations. Nauplius 26(0): e2018038. 10.1590/2358-2936e2018038

[B15] Campos-Filho IS Fernandes CS Cardoso GM Bichuette ME Aguiar JO Taiti S (2019) Two new species and new records of terrestrial isopods (Crustacea, Isopoda, Oniscidea) from Brazilian caves. Zootaxa 4564(2): 422–448. 10.11646/zootaxa.4564.2.6 31716505

[B16] Campos-Filho IS Fernandes CS Cardoso GM Bichuette ME Aguiar JO Taiti S (2020) New species and new records of terrestrial isopods (Crustacea, Isopoda, Oniscidea) of the families Philosciidae and Scleropactidae from Brazilian caves. European Journal of Taxonomy 606(606): 1–38. 10.5852/ejt.2020.606

[B17] Campos-Filho IS Gallo JS Gallão JE Torres DF Carpio-Díaz YM López-Orozco CM Borja-Arrieta R Taiti S Bichuette ME (2022a) Expanding the knowledge on the diversity of the cavernicolous Styloniscidae Vandel, 1952 (Oniscidea, Synocheta) from Brazil, with descriptions of two new species from the semiarid karst regions. ZooKeys 1101: 35–55. 10.3897/zookeys.1101.79043 36760971 PMC9848953

[B18] Campos-Filho IS Gallo JS Gallão JE Torres DF Horta L Carpio-Díaz YM López-Orozco CM Borja-Arrieta R Aguiar JO Bichuette ME (2022b) Unique and fragile diversity emerges from Brazilian caves – two new amphibious species of *Xangoniscus* Campos-Filho, Araujo & Taiti, 2014 (Oniscidea, Styloniscidae) from Serra do Ramalho karst area, state of Bahia, Brazil. Subterranean Biology 42: 1–22. 10.3897/subtbiol.42.75725

[B19] Campos-Filho IS Cardoso GM Bichuette ME (2022c) Isopoda, Oniscidea. In: Zampaulo RA Prous X (Eds) Fauna cavernícola do Brasil.Editora Rupestre, Belo Horizonte, Brazil, 363–387.

[B20] Campos-Filho IS Sfenthourakis S Gallo JS Gallão JE Torres DF Chagas-Jr A Horta L Carpio-Díaz YM López-Orozco CM Borja-Arrieta R Araujo PB Taiti S Bichuette ME (2023) Shedding light into Brazilian subterranean isopods (Isopoda, Oniscidea): Expanding distribution data and describing new taxa. Zoosystema 45(19): 531–599. 10.5252/zoosystema2023v45a19

[B21] Cardoso GM Ferreira RL (2023) New troglobitic species of *Benthana* Budde-Lund, 1908 and *Benthanoides* Lemos de Castro, 1958 from iron-ore caves and their important record in the Amazon biome (Crustacea: Isopoda: Philosciidae). Zootaxa 4(27): 548–562. 10.11646/zootaxa.5319.4.5 37518213

[B22] Cardoso GM Bastos-Pereira R Souza LA Ferreira RL (2020a) New troglobitic species of *Xangoniscus* (Isopoda: Styloniscidae) from Brazil, with notes on their habitats and threats. Zootaxa 4819(1): 084–108. 10.11646/zootaxa.4819.1.4 33055671

[B23] Cardoso GM Bastos-Pereira R Souza LA Ferreira RL (2020b) New cave species of *Pectenoniscus* Andersson, 1960 (Isopoda: Oniscidea: Styloniscidae) and an identification key for the genus. Nauplius 28: e2020039. 10.1590/2358-2936e2020039

[B24] Cardoso GM Bastos-Pereira R Souza LA Ferreira RL (2021) *Chaimowiczia*: A new Iuiuniscinae genus from Brazil (Oniscidea, Synocheta, Styloniscidae) with the description of two new troglobitic species. Subterranean Biology 39: 45–62. 10.3897/subtbiol.39.65305

[B25] Cordeiro LM Borghezan R Trajano E (2014) Subterranean biodiversity in the Serra da Bodoquena karst area, Paraguay river basin, Mato Grosso do Sul, Southwestern Brazil. Biota Neotropica 14(3): e20140114. 10.1590/1676-06032014011414

[B26] Dimitriou AC Taiti S Sfenthourakis S (2019) Genetic evidence against monophyly of Oniscidea implies a need to revise scenarios for the origin of terrestrial isopods. Scientific Reports 9(1): e18508. 10.1038/s41598-019-55071-4 PMC6898597 31811226

[B27] Erhard F (1998) Phylogenetic relationships within the Oniscidea (Crustacea, Isopoda). Israel Journal of Zoology 44: 303–309.

[B28] Ferrara F Paoli P Taiti S (1994) Philosciids with pleopodal lungs? The case of the genus *Aphiloscia* Budde-Lund, 1908 (Crustacea: Isopoda: Oniscidea), with a description of six new species. Journal of Natural History 28(6): 1231–1264. 10.1080/00222939400770631

[B29] Galati EAB Nunes VLB Boggiani PC Dorval MEC Cristaldo G Rocha HC Oshiro ET Gonçalves-de-Andrade RM Naufel G (2003) *Phlebotomines* (Diptera, Psychodidae) in caves of the Serra da Bodoquena, Mato Grosso do Sul state, Brazil. Revista Brasileira de Entomologia 47(2): 283–296. 10.1590/S0085-56262003000200017

[B30] Guzik MT Stringer DN Murphy NP Cooper SJB Taiti S King RA Humphreys WF Austin AD (2019) Molecular phylogenetic analysis of Australian arid-zone oniscidean isopods (Crustacea: Haloniscus) reveals strong regional endemicity and new putative species. Invertebrate Systematics 33: 556–574.

[B31] Javidkar M Cooper SJB King RA Humphreys WF Austin A (2015) Molecular phylogenetic analyses reveal a new southern hemisphere oniscidean family (Crustacea: Isopoda) with a unique water transport system. Invertebrate Systematics 29(6): 554–577. 10.1071/IS15010

[B32] Koenemann S Holsinger JR (1999) *Megagidiellaazul*, a new genus and species of cavernicolous amphipod crustacean (Bogidiellidae) from Brazil, with remarks on its biogeographic and phylogenetic relationships. Proceedings of the Biological Society of Washington 112(3): 572–580. https://biostor.org/reference/74272

[B33] Leistikow A (2000) Terrestrial Isopoda from Guatemala and Mexico (Crustacea: Oniscidea: Crinocheta). Revue Suisse de Zoologie 107(2): 283–323. 10.5962/bhl.part.80131

[B34] Leistikow A (2001) Phylogeny and biogeography of South American Crinocheta, traditionally placed in the family “Philosciidae” (Crustacea: Isopoda: Oniscidea). Organisms, Diversity & Evolution, Electronic Supplement 4: 1–85. 10.1078/1439-6092-00020 [Accessed 6 Feb. 2020]

[B35] Leistikow A Araujo PB (2001) Morphology of respiratory organs in South American Oniscidea (“Philosciidae”). Crustacean Issues 13: 329–336.

[B36] Lins LSF Ho SYW Lo N (2017) An evolutionary timescale for terrestrial isopods and a lack of molecular support for the monophyly of Oniscidea (Crustacea: Isopoda). Organisms, Diversity & Evolution 17(4): 813–820. 10.1007/s13127-017-0346-2

[B37] Lobo HAS (2007) Método para avaliação do potencial espeleoturístico do Parque Nacional da Serra da Bodoquena, MS. Caderno Virtual de Turismo 7(3): 99–110.

[B38] López-Orozco CM Carpio-Díaz YM Borja-Arrieta R Navas GR Campos-Filho IS Taiti S Mateos M Olazaran A Caballero IC Jotty K Gómez-Estrada H Hurtado L (2022) A glimpse into remarkable unkown diversity of oniscideans along the Caribbean coasts revealed on a tiny island. European Journal of Taxonomy 793: 1–50. 10.5852/ejt.2022.793.1643

[B39] López-Orozco CM Campos-Filho IS Gallo JS Gallão JE Carpio-Díaz YM Borja-Arrieta R Bichuette ME (2024) Iron-Isopods: New records and new species of terrestrial isopods (Isopoda, Oniscidea) from Brazilian Amazon iron ore caves. European Journal of Taxonomy 921: 116–135. 10.5852/ejt.2024.921.2421

[B40] Montesanto G (2015) A fast GNU method to draw accurate scientific illustrations for taxonomy. ZooKeys 515: 191–206. 10.3897/zookeys.515.9459 PMC4525044 26261449

[B41] Montesanto G (2016) Drawing setae: A GNU way for digital scientific illustrations. Nauplius 24(0): e2016017. 10.1590/2358-2936e2016017

[B42] Moracchioli N Trajano E (2002) Bodoquena karst area, southwest Brazil: a hotspot of biodiversity for aquatic troglobites. In: XVI^th^ International Symposium of Biospeleology, Verona, 84–84.

[B43] Moutaouakil S Boulanouar M Ghamizi M Lips J Ferreira RL (2023) Two new sympatric cave species of *Castellanethes* (Isopoda, Oniscidea, Olibrinidae) from Western High Atlas of Morocco. Subterranean Biology 45: 17–37. 10.3897/subtbiol.45.95845

[B44] Pires AMS (1987) *Potiicoarabrasiliensis*: a new genus and species of Spelaeogriphacea (Crustacea: Peracarida) from Brazil with a phylogenetic analysis of the Peracarida. Journal of Natural History 21(1): 225–238. 10.1080/00222938700770101

[B45] Reboleira ASPS Goncalves F Oromí P Taiti S (2015) The cavernicolous Oniscidea (Crustacea: Isopoda) of Portugal. European Journal of Taxonomy 161(161): 1–61. 10.5852/ejt.2015.161

[B46] Schmalfuss H (1984) Eco-morphological strategies in terrestrial isopods. The Symposium Held at the Zoological Society of London 53: 49–63.

[B47] Schmalfuss H (1989) Phylogenetics in Oniscidea. Monitore Zoologico Italiano 4: 3–27.

[B48] Schmalfuss H (2003) World catalog of terrestrial isopods (Isopoda: Oniscidea). Stuttgarter Beiträge zur Naturkunde, Serie A 654: 1–341.

[B49] Schmidt C (2002) Contribution to the phylogenetic system of the Crinocheta (Crustacea, Isopoda). Part 1 (Olibrinidae to Scyphacidae s. str.). Zoosystematics and Evolution 78(2): 275–352. 10.1002/mmnz.20020780207

[B50] Schmidt C (2003) Contribution to the phylogenetic system of the Crinocheta (Crustacea, Isopoda). Part 2 (Oniscoidea to Armadillidiidae). Mitteilungen aus dem Zoologischen Museum in Berlin, Zoologische Reihe 79: 3–179. 10.1002/mmnz.20030790102

[B51] Schmidt C (2008) Phylogeny of the terrestrial Isopoda (Oniscidea): A review. Arthropod Systematics & Phylogeny 66(2): 191–226. 10.3897/asp.66.e31684

[B52] Sfenthourakis S Taiti S (2015) Patterns of taxonomic diversity among terrestrial isopods. ZooKeys 515: 13–25. 10.3897/zookeys.515.9332 PMC4525032 26261437

[B53] Sfenthourakis S Myers AA Taiti S Lowry JK (2020) Terrestrial environments. In: Thiel M Poore G (Eds) Evolution and Biogeography of the Crustacea, the Natural History of the Crustacea.Oxford University Press, Oxford, 375–404. 10.1093/oso/9780190637842.003.0014

[B54] Souza LA Ferreira RL Senna AR (2015) Amphibious shelter-builder Oniscidea species from the New World with description of a new subfamily, a new genus and a new species from Brazilian Cave (Isopoda, Synocheta, Styloniscidae). PLOS ONE 10(5): e0115021. 10.1371/journal.pone.0115021 PMC4439081 25992909

[B55] Stringer DN King RA Taiti S Guzik MT Cooper SJ Austin AD (2019) Systematics of *Haloniscus* Chilton, 1920 (Isopoda: Oniscidea: Philosciidae), with description of four new species from threatened Great Artesian Basin springs in South Australia. Journal of Crustacean Biology 39(5): 651–668. 10.1093/jcbiol/ruz044

[B56] Tabacaru I Giurginca A (2013) Cavernicolous Oniscidea of Romania. Travaux de l’Institut de Speologie. Emile Racovitza 42: 3–26.

[B57] Taiti S (2004) Crustacea: Isopoda: Oniscidea (woodlice). In: Gunn J (Ed.) Encyclopedia of caves and karst science.Fitzroy Dearborn, Taylor and Francis Group, New York, United States, 547–551.

[B58] Taiti S Ferrara F (1980) The family Philosciidae (CrustaceaOniscoidea) in Africa, south of the Sahara. Monitore Zoologico Italiano Supplemento 13(1): 53–98. 10.1080/00269786.1980.11758549

[B59] Taiti S Ferrara F (1982) Revision of the family Philosciidae (Crustacea, Isopoda, Oniscoidea) from South Africa. Annals of the South African Museum 90(1): 1–48.

[B60] Taiti S Ferrara F (2004) The terrestrial Isopoda (Crustacea: Oniscidea) of the Socotra Archipelago. Fauna of Arabia 20: 211–325.

[B61] Taiti S Gardini P (2022) The family Olibrinidae in Italy (MalacostracaIsopodaOniscidea). Redia (Firenze) 105: 97–105. 10.19263/REDIA-105.22.13

[B62] Taiti S Gruber GA (2008) Cave-dwelling terrestrial isopods from Southern China (Crustacea, Isopoda, Oniscidea), with descriptions of four new species. In: Latella L Zorzin R (Eds) Research in South China karsts.Memorie del Museo Civico di Storia Naturale di Verona, Monografie Naturalistiche, 101–123.

[B63] Taiti S Humphreys WF (2001) New aquatic Oniscidea (Crustacea: Isopoda) from groundwater calcretes of Western Australia. Records of the Western Australian Museum 64(1): 133–151. 10.18195/issn.0313-122x.64.2001.133-151

[B64] Taiti S Xue Z (2012) The cavernicolous genus *Trogloniscus* nomem novum, with descriptions of four new species from southern China (Crustacea, Oniscidea, Styloniscidae). Tropical Zoology 25(4): 183–209. 10.1080/03946975.2012.751240

[B65] Taiti S Argano R Marcia P Scarpa F Sanna D Casu M (2018) The genus *Alpioniscus* Racovitza, 1908 in Sardinia: Taxonomy and natural history (Isopoda, Oniscidea, Trichoniscidae). ZooKeys 801: 229–263. 10.3897/zookeys.801.24102 PMC6288260 30564038

[B66] Trajano E Gallão JE Bichuette ME (2016) Spots of high diversity of troglobites in Brazil: The challenge of measuring subterranean diversity. Biodiversity and Conservation 25(10): 1805–1828. 10.1007/s10531-016-1151-5

[B67] Vandel A (1973) Les isopodes terrestres et cavernicoles de l’île de Cuba. In: Orghidan T Núñez A Botosaneanu L Decou V Negrea Ş Viña N (Eds) Résultats des Expéditions biospéologiques cubanoroumaines à Cuba, Vol.1. Editura Academiei Republicii Socialiste România, Bucharest, 153–188.

[B68] Vittori M Dominko M (2022) A bibliometric analysis of research on terrestrial isopods. ZooKeys 1101: 13–34. 10.3897/zookeys.1101.81016 36760969 PMC9848840

[B69] Wägele JW (1989) Evolution und phylogenetisches System der Isopoda. Zoologica 140: 1–262.

[B70] Warburg MR Adis J Rosenberg M Schaller F (1997) Ecology and the structure of respiratory organs in a unique amphibious/terrestrial isopod from the Neotropics (Oniscidea: Philosciidae). Studies on Neotropical Fauna and Environment 32: 52–63.

